# Genomic mosaicism in the developing and adult brain

**DOI:** 10.1002/dneu.22626

**Published:** 2018-08-01

**Authors:** Suzanne Rohrback, Benjamin Siddoway, Christine S. Liu, Jerold Chun

**Affiliations:** ^1^ Biomedical Sciences Graduate Program, School of Medicine University of California San Diego La Jolla California 92093; ^2^ Sanford Burnham Prebys Medical Discovery Institute La Jolla California; ^3^ Present address: Illumina, Inc. San Diego CA 92122 USA

**Keywords:** genomic mosaicism, somatic mutation, neurodegenerative disease, retrotransposition, reverse transcriptase, DNA damage, plasticity, memory, RNA, GWAS, sporadic

## Abstract

Since the discovery of DNA, the normal developing and functioning brain has been assumed to be composed of cells with identical genomes, which remains the dominant view even today. However, this pervasive assumption is incorrect, as proven by increasing numbers of reports within the last 20 years that have identified multiple forms of somatically produced genomic mosaicism (GM), wherein brain cells—especially neurons—from a single individual show diverse alterations in DNA, distinct from the germline. Critically, these changes alter the actual DNA nucleotide sequences—in contrast to epigenetic mechanisms—and almost certainly contribute to the remarkably diverse phenotypes of single brain cells, including single‐cell transcriptomic profiles. Here, we review the history of GM within the normal brain, including its major forms, initiating mechanisms, and possible functions. GM forms include aneuploidies and aneusomies, smaller copy number variations (CNVs), long interspersed nuclear element type 1 (LINE1) repeat elements, and single nucleotide variations (SNVs), as well as DNA content variation (DCV) that reflects all forms of GM with greatest coverage of large, brain cell populations. In addition, technical considerations are examined, along with relationships among GM forms and multiple brain diseases. GM affecting genes and loci within the brain contrast with current neural discovery approaches that rely on sequencing nonbrain DNA (e.g., genome‐wide association studies (GWAS)). Increasing knowledge of neural GM has implications for mechanisms of development, diversity, and function, as well as understanding diseases, particularly considering the overwhelming prevalence of sporadic brain diseases that are unlinked to germline mutations. © 2018 The Authors. Developmental Neurobiology Published by Wiley Periodicals, Inc. Develop Neurobiol, 2018

## INTRODUCTION

The exquisite organization and complexity of cells within the brain have been recognized since the days of Golgi and Cajal (Cajal, 1901) at the turn of the 20th century, yet molecular mechanisms from which the brain develops and functions are still not completely understood. However, in the 1940s and 1950s, the discovery of DNA as the carrier of genes (Avery et al., 1944; McCarty, 1995, 2003) and its structural implications for genetics (Watson and Crick, 1953a, 1953b) led to a model in which cells of the brain—and all other organs in an individual—arise from a single, genetic blueprint within a constant, diploid genome of unchanging sequence. Each individual has thus been generally thought to be made up of genomically identical cells, arising from a genome first formed at fertilization as a zygote and extending through mitotic cell division to all cells of the body, including the cells of the brain (*Do all our body cells have the same DNA?* 2012). A corollary of this assumption is that the universe of normal activities of the brain—from development and functional organization to personality and consciousness—arise from a single, immutable genome.

A prominent exception to the view that all cells of an individual have identical genomes emerged in the 1960s from theoretical estimates of antibody diversity vs. available genetic information (Dreyer and Bennett, 1965; Dreyer et al., 1967) wherein estimates based upon genome size indicated that there was insufficient genomic information to encode for the recognized astronomical diversity of antibodies. This realization implicated somatic changes in DNA sequence—occurring post‐zygotically—to allow gene recombination to produce new, combinatorial coding sequences. This process is known today as V(D)J recombination (Hozumi and Tonegawa, 1976; Schatz and Baltimore, 1988; Jung et al., 2006), which affects both immunoglobulin and T cell receptor loci, and further forms of somatic events within adaptive immune cells occur through heavy chain class switch recombination (Xu et al., 2012) and somatic hypermutation (Odegard and Schatz, 2006). Together, these changes underlie the enormous repertoire of antigen receptors that define the mammalian adaptive immune system. One result of somatic recombination and hypermutation is production of immune cells that form a complex mosaic composed of cells having different DNA sequences. This process occurs somatically and is thus not passed on through the germline. The immune system of an individual therefore represents a first and clear example of genomic mosaicism (GM) involving *normal* cells of the body, with clear functional consequences through the generation of antigen receptors, and selection and survival of immune cells (Surh and Sprent, 1994; Shlomchik and Weisel, 2012; Stritesky et al., 2012).

An operational definition of GM includes the following features: (1) it occurs somatically and therefore does not affect germline DNA sequences, contrasting with “genetic” mutations that enter the germline (hence the term “genomic” rather than “genetic” mosaicism here); (2) it produces nucleotide sequence changes, as differentiated from epigenetics (Wolffe and Matzke, 1999) which does not, thus allowing cells in an individual to be distinguished by their DNA sequence, and (3) it encompasses *all* forms of DNA sequence changes that include gains, losses, substitutions, and rearrangements, as well as gene recombination. Importantly, GM can clearly have functional consequences, rather than being simply an epiphenomenon, as already noted for the immune system.

Could GM contribute to the vast cell diversity of form and function within the brain? Early speculations in the 1960s used a general analogy of nervous system complexity as revealed in a tissue section from goldfish tectum (Dreyer et al., 1967) and compared it to immunoglobulin diversity; these conjectures were intriguing but not based on experimental evidence. However, unlike successful identification, in subsequent decades, of somatic DNA rearrangements and mutations in the immune system (Hozumi and Tonegawa, 1976), no definitive (or even circumstantial) evidence of GM in the normal nervous system existed in the scientific literature through 1990. The identification of components of the V(D)J cleavage complex (Schatz et al., 1989), and expression within the brain of one of its components, the recombination activating gene 1 (*RAG1*) (Chun et al., 1991), rekindled interest in the possibility that GM, potentially produced by DNA recombination, could exist within the brain, albeit distinct from V(D)J recombination in that the latter additionally requires *RAG2* expression (Chun et al., 1991). However, efforts to identify neural GM by designed recombination reporters within the brain (Matsuoka et al., 1991; Schatz and Chun, 1992) or candidate gene examinations that included olfactory receptors (Chun, 2004; Eggan et al., 2004; Li et al., 2004), protocadherins (Chun, 1999; Wu and Maniatis, 1999), and drosophila *DSCAM* (Hattori et al., 2008; Jin et al., 2013) did not support recombination or other directed mechanisms that could produce neural GM.

**Figure 1:**
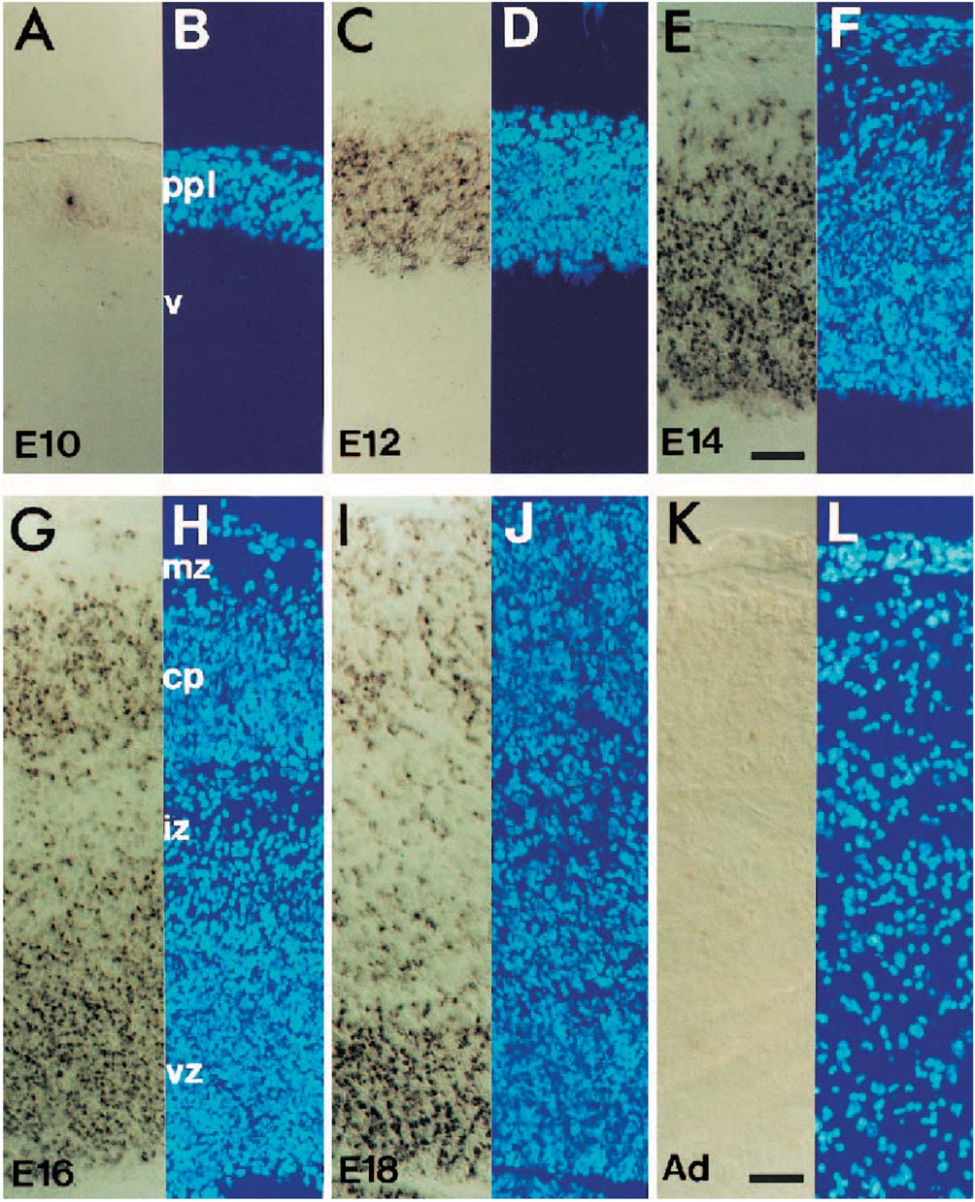
Double strand break labeling in the developing brain. **(A to L)** Nuclear DAPI staining and *in situ* end‐labeling plus (ISEL+) of double strand breaks in the embryonic mouse cortex from embryonic days 10–18 (E10‐E18) and adult (adapted from Blaschke et al., 1996). ISEL+ labeling increases through E14 and subsequently decreases with further development. [Color figure can be viewed at http://wileyonlinelibrary.com]

Commencing in the mid‐1990s, distinct approaches to GM characterization emerged from studies of programmed cell death during neurogenesis (Blaschke et al., 1996; Kuida et al., 1996; Voyvodic, 1996; Staley et al., 1997; Blaschke et al., 1998; Kuida et al., 1998; Pompeiano et al., 1998; Pompeiano et al., 2000; McConnell et al., 2009; Yung et al., 2009; Peterson et al., 2012). These approaches revealed extensive DNA fragmentation within single neurons that could precede cell death by days (Pompeiano et al., 1998), reminiscent of myriad apoptotic cells found in regions undergoing immunological gene recombination (Surh and Sprent, 1994; Chun, 2001). The intermixture of cells with varied levels of fragmented DNA amidst those without fragmentation, virtually all of which appeared normal by standard histological stains, supported tolerance of genomically distinct cells, as defined by levels of DNA fragmentation (Blaschke et al., 1996; Staley et al., 1997; Blaschke et al., 1998; Pompeiano et al., 2000, 1998) within a normal, developing brain, although having primary fates leading ultimately to cell death (Fig. 1) (Blaschke et al., 1996; Blaschke et al., 1998; Pompeiano et al., 2000). Interestingly, at least some of the DNA fragments were associated with apoptotic nucleosomal ladders that possessed blunt‐ended, 5′‐phosphorylated ends (Blaschke et al., 1996; Staley et al., 1997), as is also found during V(D)J recombination (Schlissel et al., 1993). Moreover, proteins required to join the ends during V(D)J recombination—nonhomologous end‐joining (NHEJ) proteins (XRCC4, LigIV)—were found to produce a major phenotype when deleted in mice: massive cell death within the cerebral cortex (and other regions), resulting in embryonic lethality (Chun and Schatz, 1999; Gu et al., 2000).

**Figure 2:**
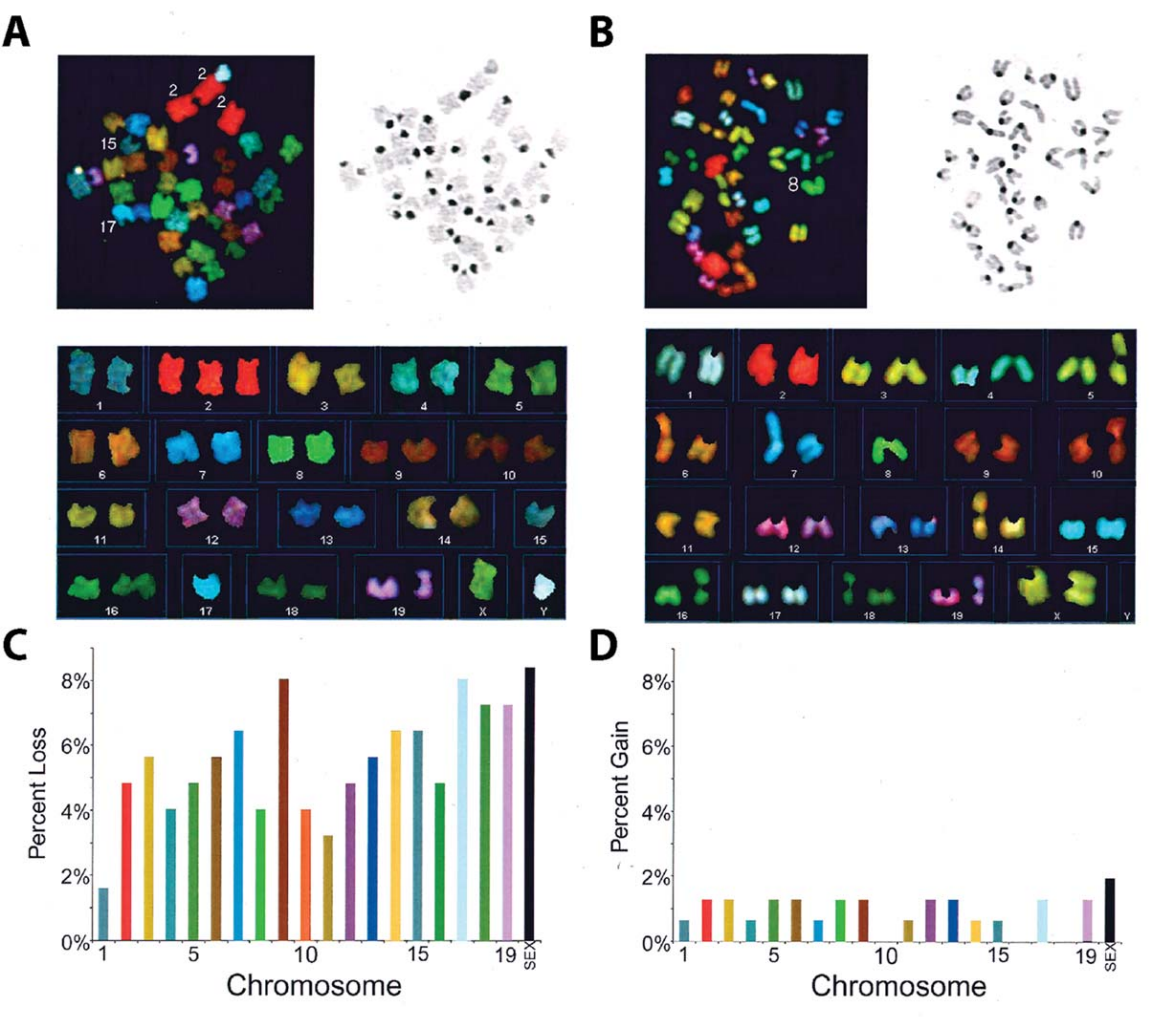
Spectral karyotyping (SKY) of NPCs in the developing brain. Spectral karyotyping (SKY) of representative mouse embryonic neuroprogenitor metaphase spreads (adapted from Rehen et al., 2001). **(A and B)** Spectral (top left) and DAPI (top right) images show chromosome spreads and unique spectral colors for each chromosome. **(C and D)** Karyotypes (bottom) illustrate losses or gains of particular chromosomes across experiments. Euploid mouse cells have two copies of each chromosome for a total of 40. [Color figure can be viewed at http://wileyonlinelibrary.com]

**Figure 3:**
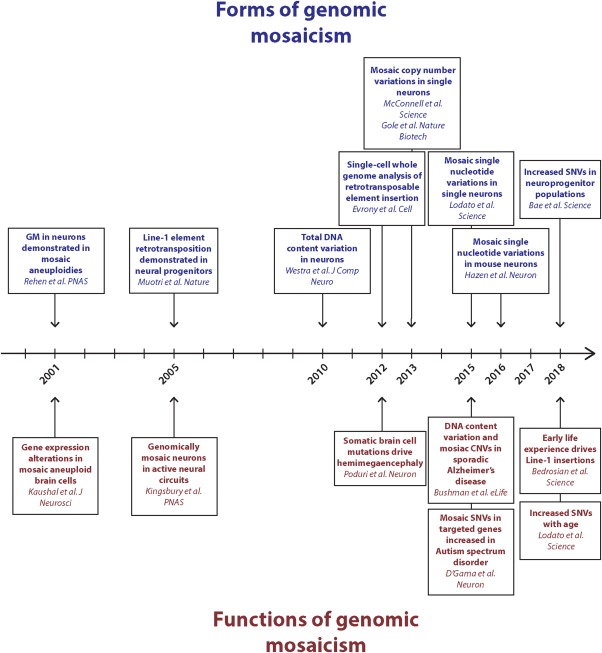
Timeline of studies identifying forms of genomic mosaicism and reported functions and/or consequences. As higher‐resolution NGS technologies have become available, the ability to identify smaller somatic differences between brain cells has improved. Many forms of GM have significant functional implications in both healthy brain and disease states. [Color figure can be viewed at http://wileyonlinelibrary.com]

Possible sequelae of NHEJ loss, including genomic instability and aneuploidy, were documented in cancers (Difilippantonio et al., 2000; Deans et al., 2003; Thacker and Zdzienicka, 2004), which led to a directed search for aneuploid cells during neurogenesis within the embryonic cerebral cortex. This approach identified the first definitive evidence of neural GM—that which occurred among cells of a single brain—through mosaic, complex aneuploidies among mitotic neural progenitor cells (Rehen et al., 2001) (Fig. 2), and also represents a first example of DNA copy number variations (CNVs). In subsequent years, other forms of GM were identified, including LINE1 elements and sub‐chromosomal CNVs, both of which can be captured by DNA content variation (DCV), as well as single nucleotide variations (SNVs), which together reveal the pervasive prevalence of GM throughout the brain (Kingsbury et al., 2006; Westra et al., 2010; Bushman and Chun, 2013; Bushman et al., 2015) (Fig. 3). The varied forms of neural GM in the normal developing and mature brain are reviewed next, followed by a discussion of functions and putative brain disease relationships.

### Genomic Mosaicism (GM) as Aneuploidy and Aneusomy in the Developing and Mature Brain

“Aneuploidy” is historically defined as the gain and/or loss of chromosomes from a euploid complement (Tackholm, 1922; Santaguida and Amon, 2015). Its study requires analyses of all chromosomes in a single cell, which, until recently, required examination of condensed chromosomes within metaphase spreads. “Aneusomy” refers typically to a copy number change of a single, defined chromosome without complete knowledge of the state of the remaining chromosomes, and it has been historically identified using fluorescence *in situ* hybridization (FISH) on nonmitotic cells (Cremer et al., 1986; Pinkel et al., 1986). Analyses of aneuploidy date back over 175 years with the discovery of chromosomes (Naegeli, 1844; Schleicher, 1879; Flemming, 1882; Waldeyer, 1888; Paweletz, 2001) and, indeed, human chromosome number was not accurately reported as 46 chromosomes until 1956 (Tjio and Levan, 1956), before which it was misidentified as 48. This error underscores the technical challenges associated with defining chromosomes before using metaphase spreads, the current gold standard, to examine chromosome number.

Using what was a new technology—spectral karyotyping (SKY) (Liyanage et al., 1996; Macville et al., 1997)—to examine aneuploidy in metaphase spreads, mitotic spreads of neural progenitor cells (NPCs) revealed pervasive aneuploidies within the developing mouse cerebral cortex, affecting ∼30% of mitotic NPCs (Fig. 2) (Rehen et al., 2001). Aneusomic postmitotic neurons (and glia) from both mouse and human were subsequently identified using SKY and/or FISH (McConnell et al., 2004; Kingsbury et al., 2005; Rehen et al., 2005; Yurov et al., 2007a; Peterson et al., 2012). In the adult brain, aneusomies constituted a range that averaged ∼18% (Yurov et al., 2005; Iourov et al., 2006, 2009ab; Yurov et al., 2008; Faggioli et al., 2012; Yurov et al., 2014) (Table 1, Human; Table 2, Mouse). That neural aneuploidies exist is supported not only by hybridization‐based techniques like SKY, but also by histological identification and real‐time imaging of lagging chromosomes and supernumerary centrosomes resulting in multipolar cell divisions, as well as by cytological identification of nondisjunction, micronuclei, and multiple nuclei (Yang et al., 2003). More recently, aneuploidies have been identified by next generation sequencing (NGS) methods (McConnell et al., 2013; Cai et al., 2014; Knouse et al., 2014; van den Bos et al., 2016). All chromosomes contribute to aneuploidy, and there does not appear to be a preference for any one chromosome being affected (Rehen et al., 2001; McConnell et al., 2004; Faggioli et al., 2012; Peterson et al., 2012; Yurov et al., 2014).

**Table 1:** Estimates of Aneuploidy Rates in the Human Cerebral Cortex

Method	Study	Age	Cell Type	Chromosome	Aneuploidy Rate^a^	Hypoploidy : Hyperploidy Ratio
FISH	Yurov et al., 2007	8–12 gestational weeks	NA	1,9,15–18, X/Y	1.5%^b^ **(29%)**	5 : 2
	Rehen et al., 2005	2 years	NA	21	3.2% **(53%)**	3 : 7
			Neuron	21	3.4% **(55%)**	7 : 9
		15 years	NA	21	3.8% **(59%)**	5 : 9
		35 years	NA	21	3.6% **(57%)**	3 : 7
			Neuron	21	2.8% **(48%)**	1: 1
		48 years	NA Neuron	21 21	3.6% **(57%)** 2.3% **(41%)**	1 : 3 5 : 7
	Iourov et al., 2009b	∼25 years	NA	13,18,21,X/Y	0.5%,0.6%,0.4%,0.3% **(10%)**	8 : 9^b^
			NA	1,11,17,18,X	0.5%,0.7%, 0.5%, 0.8%,0.4% **(13%)**	7 : 4^b^
	Iourov et al., 2009a	∼25 years	NA	1,7–9,11,16–18,X/Y	0.5%,0.7%,0.9%,0.7%,0.7%,0.5%,0.5%,0.8%,0.4% **(14%)**	4 : 7^b^
	Yurov et al., 2005	Adult	NA	1, 13/21, 18, X/Y	0.4%, 0.3%, 0.7%, 0.8% **(12%)** b	5 : 4^c^
	Yurov et al., 2008	∼60 years	NA	1	0.6% **(13%)**	1 : 1
		∼79 years	NA	1,11,17,18,X	0.6%,0.8%,0.8%,1.1%,1.4% **(20%)**	2 : 1^b^
	Yurov et al., 2014	69–82 years	NA	1,7,11,16–18	0.5%,0.7%,0.7%,0.6%,0.6%,0.8%,1.3% (**16%)**	NR
			NA	X	1.2% **(24%)**	NR
MCB	Iourov et al., 2009a	∼25 years	NA	1,7,14,21,X	0.3%,0.6%,0.4%,0.5%,1.2% **(13%)**	NR
	Iourov et al., 2006	Adult	NA	1, 9, 16, 18, X	0.3%, 0.5%, 0.4%, 0.2%, 2.0% **(15%)** b	NR
	Iourov et al., 2009b	∼25 years	NA	7,14,21,X	0.6%,0.4%,0.9%,1.2% **(16%)**	NR
		∼79 years	NA	7,14,21,X	1.0%,0.8%,1.3%,1.9% **(25%)**	NR
scWGS	McConnell et al., 2013	20–26 years	Neuron	All	**1.8%**	1 : 1
	Cai et al., 2014	42 years	Neuron	All	**4.9%**	4 : 1
	Knouse et al., 2014	40–70 years	Neuron	All	**2.2%**	1 : 1
	van den Bos et al., 2016	69–93 years	Neuron	All	**0.7%**	2 : 5

NR, not reported; all tissue samples contain neurons and all sample sizes are greater than 50 cells.

a Per chromosome frequency for FISH. Total frequency of aneuploid cells—extrapolated for FISH—is in bold.

b Averaged from multiple chromosome measurements.

c Estimated from female X chromosome only.

**Table 2:** Estimates of Aneuploidy Rates in the Mouse Cerebral Cortex

Method	Study	Age	Cell Type	Chromosome	Aneuploidy Rate^a^	Hypoploidy : Hyperploidy Ratio
FISH	Rehen et al., 2001	E13‐E14	NA	X/Y	6.7% **(80%)**	15 : 2
	Peterson et al., 2012	E19	NA	8,16	1.6%,2.1% **(31%)**	NR
	McConnell et al., 2004	8–14 weeks	NA	X/Y	6.2%^b^ **(72%)**	9 : 5^c^
	Rehen et al., 2001	Adult	NA	X/Y	1.2% **(21%)**	5 : 1
	Faggioli et al., 2012	4 months	NA	1,7,14–16,18,19 Y	1%^b^ **(18%)**	1 : 1^c^
		15 months	NA	18	1.5% **(26%)**	NR
		28 months	NA	1,7,14–16,18,19 Y	2.3%^b^ **(37%)**	1 : 1^c^
			Neuron	18	2.1% **(35%)**	NR
			Neuron	18	9.8% **(87%)**	NR
scWGS	Knouse et al., 2014	Adult	Neuron	All	**1.4%**	0 : 1
Spread counts	Peterson et al., 2012	E14	NA	All	**29.0%**	11 : 1^c^
			NA	All	**24.0%**	7 : 1^c^
SKY	McConnell et al., 2004	E12.5‐E14.5	NA	All	**34.0%** b	3 : 1^c^
	Rehen et al., 2001	Embryonic	NA	All	**33.2%**	6 : 1^b^, ^c^

NR, not reported; all tissue samples contain neurons and all sample sizes are greater than 50 cells.

a Per chromosome frequency for FISH. Total frequency of aneuploid cells—extrapolated for FISH—is in bold.

b Averaged from multiple animals or from multiple chromosomes.

c Estimated from a figure.

Neural aneuploidy is most commonly seen as hypoploidy rather than hyperploidy (chromosome loss and gain, respectively) (Rehen et al., 2001; Rehen et al., 2005; Yurov et al., 2005; Yurov et al., 2007a; Westra et al., 2008). This is consistent with the preference for segregation defects involving lagging chromosomes and supernumerary centromeres (as opposed to nondisjunction), which favor the production of hypoploidies in the developing brain (Yang et al., 2003). This difference is most dramatic when assessing the population of cycling neural progenitor cells (∼sixfold more hypoploidies than hyperploidies) (Rehen et al., 2001; McConnell et al., 2004; Peterson et al., 2012), which may suggest that there is negative selection pressure during differentiation, ultimately producing interphase, G_0_ cells with a preference for chromosome loss. An important technical aspect of assessing the prevalence of aneusomic cells in brain tissue sections is the problem of sectioning through a nucleus to render it artifactually hypoploid. For this reason, a number of studies purposefully biased examination to only chromosome gains (hypersomies) since they would not be produced by sectioning artifact, combined with a focus on sex chromosomes that provided positive, internal controls for identifying 1 (single X‐ and Y‐) chromosome in males or 2 (X‐) chromosomes in females in the face of aneuploid numbers of sex chromosome or autosome copies (Rehen et al., 2001; Kingsbury et al., 2005; Rehen et al., 2005).

Aneusomic neurons survive into adulthood (Rehen et al., 2005; Faggioli et al., 2012; McConnell et al., 2013; Cai et al., 2014; Knouse et al., 2014) where they can become integrated as active components of neuronal circuitry (Kingsbury et al., 2005), and thus likely have functionality. As aneuploid cells are known to have altered gene expression patterns (Yang et al., 2003; Sheltzer et al., 2012), this likely contributes to functional diversity (Letourneau et al., 2014). In addition, although the majority of studies have focused on the cerebral cortex, aneuploidy or aneusomy appears to be ubiquitous across the neuraxis, and has been reported within the medulla oblongata (Yurov et al., 2005), cerebellum (Westra et al., 2008; Iourov et al., 2009a; Faggioli et al., 2012), entorhinal cortex (Mosch et al., 2007), and hippocampus (Rehen et al., 2005; Yurov et al., 2014). Although these tissues are less thoroughly characterized, they generally appear to have similar levels of aneuploidy as the cortex, although there is some evidence that the hippocampus may have slightly higher (Rehen et al., 2005; Yurov et al., 2014) and the cerebellum lower (Faggioli et al., 2012) rates. This form of GM extends evolutionarily from humans through at least teleost fish (Rajendran et al., 2007).

### GM as Long Interspersed Nuclear Element 1 (LINE1) Retrotransposons

The second identified element capable of producing GM was LINE1 elements. Retrotransposable elements have produced genomic diversification in both evolution and cancer (Cordaux and Batzer, 2009; Lee et al., 2012). In humans, approximately 17% of the genome is composed of LINE1 repetitive elements within the germline (Viollet et al., 2014), where they exist as over 500,000 copies, most of which are thought to be inactive evolutionary remnants, owing, in part, to many stop codons within their ∼6 Kb sequence (Hancks and Kazazian, 2012). The bicistronic LINE1 RNA contains 2 open reading frames—*ORF1* and *ORF2*—that encode proteins. *ORF1* is thought to encode a high affinity RNA binding protein and *ORF2* a reverse transcriptase and endonuclease, which together can allow LINE1 DNA insertion into a new genomic location (Hancks and Kazazian, 2012). This process is analogous to the integration of retroviral proviruses (Varmus, 1982) except for a lack of long terminal repeat (LTR) flanking sequences. Mosaic LINE1 insertions, like other elements producing GM, have been hypothesized to contribute to neuronal diversity (Muotri et al., 2005) through somatic retrotransposition into the genome in neuronal precursors from rat hippocampus neural stem cells (Muotri et al., 2005). Further studies expanded the characterization of these elements to human neural stem cell lines that also reported higher copies of LINE1 elements in neural cell populations in comparison to other tissues (Muotri et al., 2009). A variety of sequencing approaches have supported the presence of *de novo* LINE1 insertions, albeit with widely ranging estimates of their prevalence: <0.6 per genome (Evrony et al., 2012), along with more controversial levels of ∼14 per genome, while other repeat elements (ALUs and STRs) have also been reported and debated (Baillie et al., 2011; Upton et al., 2015; Evrony et al., 2016).

### GM Produced by DNA Content Variation (DCV)

**Figure 4:**
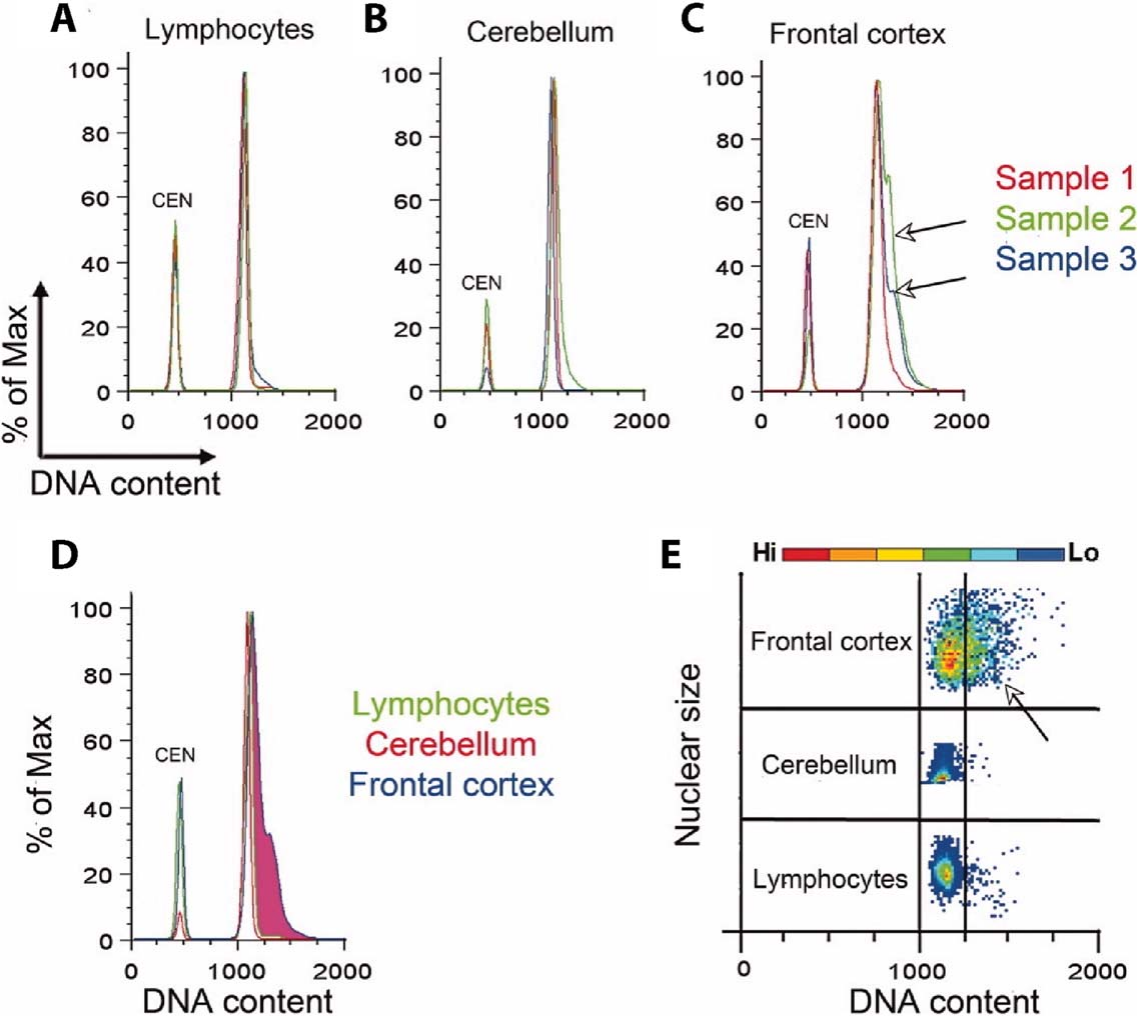
DNA content analysis of nondiseased human nuclei from brain and lymphocytes (adapted from Westra et al., 2010). Chicken erythrocyte nuclei (CEN) were included in each sample as an internal control. **(A and B)** Histograms of lymphocyte and cerebellar samples do not indicate any increase in genomic content. **(C and D)** Analysis of DNA content in frontal cortex nuclei has a broad “shoulder” to the right of the main peak revealing increased DNA content. (**E)** This increase can also be visualized in a flow cytometry plot of individual nuclei. [Color figure can be viewed at http://wileyonlinelibrary.com]

The third form of neural GM to be reported was termed DCV. This was first detected in human brain by a combination of brain cell nuclei isolation, labeling with fluorescent DNA dyes (with removal of RNA by digestion), and analyses by either flow cytometry or fluorescence activated cell sorting (FACS) (Westra et al., 2010). A marked population of cells with DNA content gain, as well as populations with loss, were observed, suggesting a further example of CNVs in addition to aneuploidies, manifesting as DCV. Importantly, this approach enabled interrogation of orders of magnitude more cells in a single experiment to reveal major population changes in the total genomic DNA of individual cells interrogated by flow cytometry/FACS (Fig. 4). DCV analyses of nuclei from postmortem human (as well as mouse) brain revealed that many, and in some cases a majority of, neurons, particularly within the human prefrontal cortex, contain more DNA than do lymphocyte controls, which contain, on average, nearly 250 Mb. The technical approach was further optimized to use the now common technique of separating neuronal nuclei based upon their NeuN‐immunopositivity by FACS (or FANS: fluorescence activated nuclear sorting). This technique was first developed and reported in earlier studies of GM (Rehen et al., 2005), in which the most prominent DCV gains occurred in neurons (Westra et al., 2010; Bushman et al., 2015). Moreover, DCV varied with neuroanatomical location, being limited in the cerebellum from the same brain, and reduced in some cell types, as seen in NeuN‐negative populations that were more similar to nuclei isolated from lymphocytes. The specific DNA sequences accounting for DCV remain unknown, but it is likely that DCV comprises “large” forms of GM, like the aneuploidies/aneusomies discussed above, as well as smaller variations broadly dispersed throughout the genome and including LINE1 elements and sub‐chromosomal CNVs, which are discussed next.

### GM Produced by Copy Number Variations (CNVs)

GM produced by mosaic aneuploidies/aneusomies and DCV within the brain was proposed to include DNA sequence changes such as mosaic CNVs with neuroanatomical region specific patterns (Westra et al., 2010). The existence of mosaic neural CNVs was further supported by FISH studies using chromosomal point probes that detected only small chromosomal regions, and may, therefore, have reported CNVs in addition to complete gain and/or loss of an entire chromosome. This phenomenon likely explains, in part, why FISH studies often report higher levels of aneusomy than methods which capture information about entire chromosomes (Knouse et al., 2014; van den Bos et al., 2016). Mosaic CNVs became possible to interrogate more comprehensively using the technical development of single‐cell whole genome sequencing (scWGS), an approach that, like many aspects of DNA sequencing, remains a work in progress. The first report of neuronal scWGS (Evrony et al., 2012) in fact did not report CNVs, but was targeted toward identifying novel LINE1 insertions. However, subsequent reports have identified a range of somatic, neural CNVs focused primarily on neurons (Gole et al., 2013; McConnell et al., 2013; Cai et al., 2014; Knouse et al., 2014; Knouse et al., 2016; van den Bos et al., 2016). To date, at least four publications using scWGS have reported the presence of somatically derived mosaic CNVs in human neurons (Table 3). Mosaic CNVs in these studies showed wide variability, reporting between 9 and 100% of neurons as containing CNVs, with most reported alterations of between 2 and 10 Mb in size, and deletions far outnumbering amplifications (Gole et al., 2013; McConnell et al., 2013; Cai et al., 2014; Knouse et al., 2016), as had been observed earlier for more common chromosomal hypoploidies. In contrast, skin or fibroblast cells were estimated to contain 0.2–0.3 CNVs per cell, with fewer than 25% of cells having any, indicating that this form of GM is enriched in the brain (McConnell et al., 2013; Knouse et al., 2016). Current efforts are ongoing to improve the specificity of this characterization and better understand the developmental relevance of neural CNVs (Rohrback et al., submitted). Still to be determined are the functional consequences of CNVs within the brain.

**Table 3:** Somatic CNV Characteristics

Study	Methods (Amplification, CNV Calling)	QC Metrics	Age (years)	Cells Analyzed^a^	Smallest Detectable CNV	CNVs per Cell^a^	Deletion : Amplification Ratio^a^	CNV Positive Cell Frequency^a^
McConnell et al., 2013	WGA4, CBS	Reads, MAD, CS	20, 24, 26	110 (50, 19, 41)	3.4 Mb	1.3 (2.1, 0.9, 0.6)	2 : 1 (3:2, 8:1, 5:1)	41% (60%, 32%, 22%)
Gole et al., 2013	MDA, CBS	NR	NR	6 (4, 2)	1 Mb	6.0 (6.8, 4.5)	2 : 3 (4:5, 1:4)	100%
Cai et al., 2014	WGA4, CBS	MAPD	42	19	2.2 Mb	3.4	65 : 1	68%
Knouse et al., 2016	WGA4, CBS & HMM	VS	48, 52, 68, 70	80 (17, 22, 21, 20)	5 Mb	0.2 (0, 0.1, 0.1, 0.6)	15 : 0	9% (0%, 9%, 5%, 20%)

NR, not reported; CBS, circular binary segmentation; HMM, hidden Markov model.

a Parenthetical values indicate measurements from individual brains.

### GM Produced by Single Nucleotide Variations (SNVs)

The smallest form of somatic DNA sequence change is a SNV that can be identified by single‐cell whole genome sequencing of vastly amplified genomes combined with median 30X sequencing coverage, which has revealed SNVs between individual neurons at the level of single nucleotides (Lodato et al., 2015; Bae et al., 2018; Lodato et al., 2018). A crucial initial step in these investigations is massive amplification of single‐cell genomic DNA through use of techniques like “multiple displacement amplification” (MDA) that employs phi29 DNA polymerase, followed by high coverage, whole genome sequencing with paired end Illumina reads, which has revealed SNVs in neurons of the brain. In addition to enormous levels of amplification, data processing must informatically take into account the error rate of the utilized phi29 polymerase, chimeric artifacts, amplification bias and errors, and the significant failure rates of single genome amplifications that likely exclude neurons with genomic attributes that interfere with amplification (e.g., strand breaks, large structural variations, chromatin states). Of further note, the unpredictably biased amplification inherent to MDA generates excessive noise which precludes the reliable examination of larger structural variations such as CNVs. Nonetheless, these high depth single neuron genomic sequences have expanded the forms of GM to thousands of SNVs within single neuronal genomes that differ from the germline. High depth sequencing of single neuron SNVs produced during neurogenesis has also been used for lineage mapping of clonal populations in the adult brain (Evrony et al., 2012; Lodato et al., 2015).

An independent methodology for assessing SNVs utilized somatic cell nuclear transfer (SCNT) and mouse cloning techniques involving mitral cells of the olfactory bulb to amplify single neuronal genomes, which also identified hundreds of SNVs within seven single neurons (Hazen et al., 2016). This methodology allows high depth sequencing without artificial template amplification. It does face intrinsic limitations, including low throughput, high failure rates of SCNT, low rates of mitotic growth of the newly created cells, incompatibility with humans in requiring the use of laboratory mice, and in some cases a need to generate cloned mice, a process that likely excludes interrogation of cells with highly altered genomes (e.g., aneuploid neurons). Nevertheless, these results demonstrated that individual mitral neurons contain hundreds of unique SNVs, and considering the relatively shorter lifespan of mice vs. humans, the numbers of SNVs in mice are generally consistent with the thousands observed in older human neurons in which SNVs appear to increase with age (Bae et al., 2018; Lodato et al., 2018), albeit based upon very few neurons assessed with all of these techniques.

### GM Technical Challenges

The study of neural GM has been strongly influenced by technical advances. Metaphase spread analyses have been used to detect chromosomal abnormalities for over 100 years and are still in use today. However, it has two considerable shortcomings for in depth analysis of neural GM. First, metaphase spreads require the presence of mitotic cells, which represent a small fraction of brain cells (Blaschke et al., 1996; Blaschke et al., 1998). Second, genomic resolution is limited to large alterations that do not inform on specific DNA sequences. DNA content changes identified by use of fluorescent DNA dyes combined with flow cytometry or FACS have been widely used as a gold standard in studying the cell cycle and in plant biology (Darzynkiewicz et al., 2004; Dolezel et al., 2007) and have provided a high throughput, albeit low resolution, assessment of GM (Westra et al., 2010; Bushman et al., 2015). Flow cytometric assessments are amenable to nonmitotic analyses of DCV and do not require metaphase spreads, allowing interrogation of hundreds of thousands of nuclei from any tissue type in minutes (Westra et al., 2010; Bushman et al., 2015).

**Figure 5:**
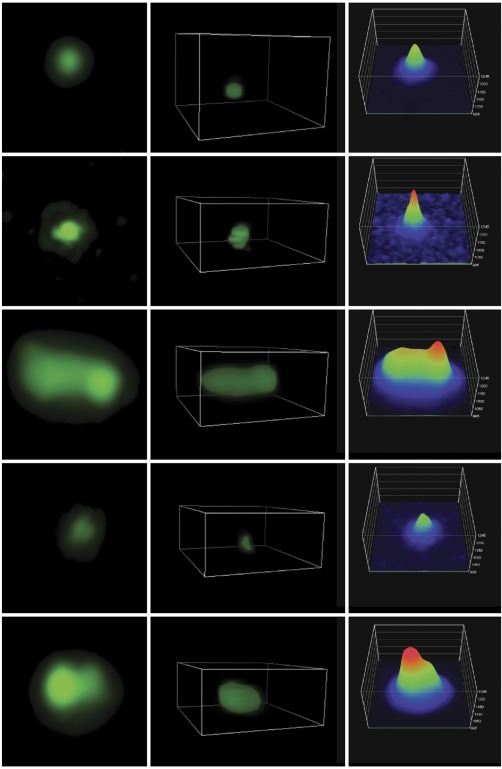
Peptide nucleic acid visualization of *APP* CNVs. Peptide nucleic acid fluorescent *in situ* hybridization was imaged using structured illumination microscopy (adapted from Bushman et al., 2015), revealing copy number variations of *APP* in Alzheimer's disease cortical neurons. [Color figure can be viewed at http://wileyonlinelibrary.com]

FISH methods, including SKY, allow a more targeted investigation of alterations to one or more chromosomes, and can be performed on mitotic, interphase, or nonmitotic cells. However, these studies have provided exceptionally variable estimates of chromosome alteration rates—from 10% to 80% of cells being aneuploid (Rehen et al., 2001; McConnell et al., 2004; Rehen et al., 2005; Yurov et al., 2005; Iourov et al., 2006, 2009ab; Yurov et al., 2007a; Yurov et al., 2008; Faggioli et al., 2012; Peterson et al., 2012; Yurov et al., 2014). A substantial amount of this variability stems from experiments performing FISH and estimating rates based on a single chromosome (Rehen et al., 2001; McConnell et al., 2004; Pack et al., 2005; Yurov et al., 2008; Faggioli et al., 2012; Yurov et al., 2014). Since metaphase segregation defects can affect multiple chromosomes simultaneously (Yang et al., 2003), aneuploidy of different chromosomes is not necessarily an independent occurrence. Thus, extrapolation produces an artificially high aneuploidy rate when an insufficient number of chromosomes are interrogated. This effect may be further compounded by the confounding variable of these methods reporting subchromosomal alterations with the same signal as for a full aneuploidy (Osada et al., 2002; Iourov et al., 2013; Bushman et al., 2015; Evrony et al., 2015; Lodato et al., 2015; Zhang et al., 2015). Peptide nucleic acid FISH (Westra et al., 2010; Bushman et al., 2015) (PNA‐FISH) provides semi‐quantitative data on a targeted locus and has the ability to visualize single genes when combined with appropriate microscopic techniques. This method has been used to identify centromere and gene copy increases (Bushman et al., 2015) *without* template amplification or other polymerase dependent approaches, and can provide validation for stereotyped CNVs reported by sequencing (Fig. 5). Additional FISH approaches based upon variations of RNAscope used in validating transcriptomic diversity in the human brain (Lake et al., 2016; Lake et al., 2017; Lake et al., 2018) may have further applicability to genomic loci in the future.

**Figure 6:**
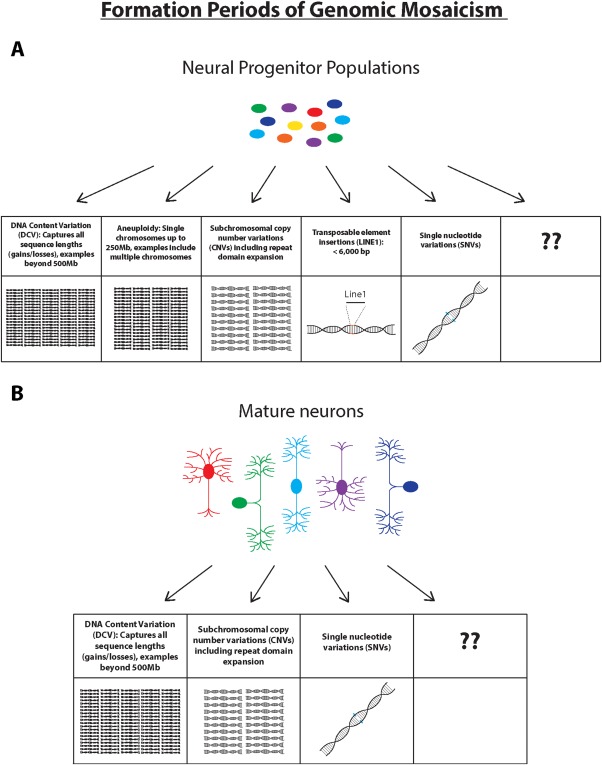
Types of GM occurring in neural progenitor populations of the developing brain and in mature neurons of the adult brain. **(A)** Multiple different forms of GM have been observed to occur in neural progenitor populations, ranging in size from over 500 Mb or more, down to the level of single nucleotide variations. **(B)** Mature neurons display increases of altered genomic sequences through at least DNA content variation (DCV), copy number variations (CNVs), and single nucleotide variations (SNVs) during postnatal life. Boxes labeled with question marks reference the current state of the field, since a range of additional forms of GM likely will be found as emerging technologies allow for new insight and novel discoveries. [Color figure can be viewed at http://wileyonlinelibrary.com]

Two approaches allow high resolution sequence information to be obtained from special treatment of bulk (multi‐cell) samples. As noted above, the first was achieved by SCNT and clonal expansion of a single neuron—where all derivative cells have identical genomes—which allowed the collection of high resolution, whole genome coverage data, albeit with the previously noted limitations. A second approach involves capture of targeted genomic regions using bulk DNA combined with pulldown “bait” strategies. The smaller size of the genome under interrogation allows ultra high depth sequencing (> 1,000×), which enables detection of somatic variants with lower allele frequency (Sala Frigerio et al., 2015) (∼1% for SNVs, 10% for CNVs). This approach is useful for known genomic targets and semi‐conserved GM alterations, but is not appropriate for *de novo* discovery or the detection of ultra low frequency somatic events as can occur with neural GM.

scWGS addresses some shortcomings of the bulk approaches, as it directly assesses the cell of interest without requiring totipotency or conservation of GM. One intriguing discrepancy has been brought to light by this method: scWGS reports much lower rates of aneuploidy (Knouse et al., 2014; van den Bos et al., 2016). This may be due to at least two factors. First, these studies have performed scWGS on interphase cells, contrasting with over 100 years of literature that has relied on definitions of aneuploidy based almost solely on metaphase spreads. Since the relative rates of aneuploidy reported in metaphase spread analyses represent a cell population that has not been assessed by scWGS, the reported discrepancies could simply reflect differences in mitotic and interphase brain cells. Second, scWGS can distinguish between full and partial chromosome alterations, while other approaches may report a broader range of aberrations that manifest in metaphase as aneuploidy. This ultimately brings into question definitions of aneuploidy in nonmitotic cells revealed by scWGS. It is notable that the relationship between interphase (nonmitotic) partial aneuploidies and chromosomes seen in condensed metaphase spreads of the brain is not known. However, SCNT approaches to condense postmitotic neuronal DNA have reported rates of aberrant chromosomal spreads of ∼64%, supporting the interpretation that partial aneuploidies/CNVs observed in nonmitotic cells—postmitotic neurons—manifest as chromosomal aneuploidies in metaphase spreads (Osada et al., 2002).

In addition to large DNA alterations, the high coverage data generated by extreme amplification using MDA and high depth sequencing allowed identification of unique SNVs (Lodato et al., 2015; Lodato et al., 2018). However, this form of scWGS has a high failure rate (possibly excluding neurons with unique genomic attributes), is cost prohibitive for more than a few cells, and regional genome bias under the reported conditions using MDA precludes examination of large structural variants such as CNVs. Lower depth sequencing following PCR‐based amplification allows analysis of CNVs, but these studies have reported extremely inconsistent findings, which is likely due to substantial methodological variability: different ages, amplification methods, sample sizes, informatics, stringencies for quality control (QC), and CNV calling, which were applied in each study (Gole et al., 2013; McConnell et al., 2013; Cai et al., 2014; Knouse et al., 2016).

At least seven critical issues associated with all scWGS approaches to assess CNV and SNV GM exist: (1) an absolute requirement to amplify the single‐cell genomic template using polymerases, which itself may introduce a range of artifacts that could create or obfuscate mosaically present CNVs or SNVs; (2) different protocols and amplification techniques—including different DNA polymerases—limit direct comparisons; (3) an inability to replicate the results, since each single cell is destroyed by sequencing; (4) a current inability to assess sub‐megabase CNVs; (5) varied and often opaque technical details that obfuscate both failure rates and bioinformatics pipeline details; (6) limited individual brain interrogation that consists of a few or even just 1 brain; and (7) miniscule sample sizes—typically less than 100 cells. Considering the 1 trillion cells in the human brain, these samples constitute only 10^−8^% of cells within a single brain.

### GM Generation, Functions, and Disease Implications

The relative sizes and forms of GM (Fig. 6) underscore a vast range—on the order of 8 logs—of DNA sequence changes, from SNVs to hundreds of millions of base pairs in a single chromosome.

Despite extensive characterization of the occurrence of neural GM, there is limited understanding of the mechanisms through which it is generated. Mosaic neural aneuploidies arise from mitotic errors that include lagging chromosomes, micronuclei, supernumerary centrosomes and chromosomal nondisjunction (Yang et al., 2003). These mechanisms are limited to periods of neurogenesis, particularly during brain development (Rehen et al., 2001; Rehen et al., 2005; Yurov et al., 2005; Westra et al., 2008). In addition to roles in generating full aneuploidies, micronuclei could contribute to smaller (megabase) CNVs. Micronuclei are a common byproduct of mitotic segregation defects and have been associated with further degradation of the affected chromosome(s) upon progression through subsequent cell cycles (Zhang et al., 2015). It is tempting to speculate that this could be one mechanism by which such CNVs are created, particularly those that appear to follow a pattern similar to chromothrypsis (McConnell et al., 2013; Cai et al., 2014; Knouse et al., 2016). Another possible source of CNVs may reflect repair of DNA, which is supported by the presence of both documented DNA breaks and nucleotide incorporation (e.g., BrdU or ^3^H‐thy) (Blaschke et al., 1996; Blaschke et al., 1998). Also proposed to occur during neurogenesis, mosaic LINE1 insertions, as discussed in a previous section, are theoretically capable of generating GM during the cell cycle (Packer et al., 1993; Muotri et al., 2005; Shi et al., 2007; Singer et al., 2010; Viollet et al., 2014; Mita et al., 2018). By contrast, many neural somatic SNVs have been associated with damage due to transcriptional activity (Lodato et al., 2015), consistent with increased SNV rates in aged brains (Bae et al., 2018; Lodato et al., 2018), suggesting this form of GM is generated in postmitotic neurons. It is entirely possible that other mechanisms could contribute to neural GM, including hypothesized gene recombination, which awaits further investigation.

The normal functions of neural GM of any form are incompletely known, yet almost certainly affect both development and adult brain function. Normal, mosaically aneuploid brain cells produce altered transcriptomes (Kaushal et al., 2003) consistent with studies in simpler systems like yeast (Sheltzer et al., 2012). In this vein, a remarkable degree of transcriptomic diversity has emerged from single‐cell transcriptome studies throughout the neuraxis of mouse (Usoskin et al., 2015; Zeisel et al., 2015; La Manno et al., 2016; Poulin et al., 2016; Tasic et al., 2016; Chung et al., 2017; Karlsson and Linnarsson, 2017; Hochgerner et al., 2018; Rosenberg et al., 2018) and the human brain (Lake et al., 2016; Lein et al., 2017; Regev et al., 2017; Sousa et al., 2017), consistent with the enormous neural GM diversity present in both mice and humans. Transcriptomic variation can cover the gamut of cellular functions, which remain to be fully assessed but are clearly part of the normal brain's circuitry, based upon the functional integration of aneusomic neurons within the brain (Kingsbury et al., 2005). During brain development, clear associations between aneuploidies and cell survival have also been documented through analyses of aneuploid neural cells following cell death attenuation by knockout of caspase 3 or caspase 9, or pan‐caspase pharmacological inhibition by Z‐VAD‐fmk (Peterson et al., 2012), which results in maintenance of increased numbers of aneuploid neural cells, including subpopulations with extreme aneuploidy that are not seen in the wildtype brain (Peterson et al., 2012). These data indicate that forms of neural aneuploidy are not neutral, with mild forms preferentially surviving, whereas more extreme forms are eliminated by cell death. For surviving postmitotic neurons (Kingsbury et al., 2005; Rehen et al., 2005), functional consequences could be vast and difficult to predict. However, based on a meta‐analysis in yeast, aneuploidy often triggers expression of stress response genes, and suppresses cell proliferation pathways (Sheltzer et al., 2012) that may relate in some instances to the postmitotic state of neurons. Possible functions of LINE1 retrotranspositions have been centered around behavior and memory (Singer et al., 2010; Bachiller et al., 2017); however germline changes in LINE1 genomic regions (Erwin et al., 2016) distinct from retrotransposition complicate analyses, which may explain major discrepancies in the literature (e.g., LINE1 putative somatic retrotransposition rates of <0.6 per genome (Evrony et al., 2012) vs. ∼14 per genome (Baillie et al., 2011; Upton et al., 2015)). It is certain that the functional consequences of neural GM will be revealed in increasingly greater detail by ongoing research.

Indeed, clear precedence for functional consequences of GM affecting the brain and body exist in data on diseased states. Mosaic variegated aneuploidy (MVA), in which inactivating gene mutations in mitotic proteins causes an increase in forms and frequency of aneuploidy, ultimately results in microcephaly and mental retardation (Warburton et al., 1991; Kajii et al., 1998). Constitutive aneuploidies as found in Down syndrome (Wiseman et al., 2015) have clear effects on brain function, providing support for functional consequences of neural mosaic aneuploidies and GM, while constitutive cases may also themselves be chromosomal mosaics (Modi et al., 2003; Leon et al., 2010; Hulten et al., 2013). Indeed, multiple reports of elevated levels of somatic aneuploidy in patients with ataxia telangiectasia (McConnell et al., 2004; Iourov et al., 2009a, 2009b), schizophrenia (Yurov et al., 2001; Yurov et al., 2008), autism (Yurov et al., 2007b; Iourov et al., 2008), and Alzheimer's disease (Pack et al., 2005; Mosch et al., 2007; Iourov et al., 2009b; Iourov et al., 2011; Yurov et al., 2014) support pathogenic links. Brain disease relationships of LINE1 GM have been reported in rare disorders resulting from mutations in genes that regulate LINE1: Rett Syndrome (*MECP2*), ataxia telangiectasia (*ATM*), and Aicardi‐Goutières syndrome (involving multiple different genes, including *SAMHD1* that inhibits viral and LINE1 reverse transcriptases). MECP2 is a suppressor of LINE1 transcription (Skene et al., 2010; Muotri et al., 2010), while ATM recognizes LINE1 target priming retrotransposition intermediates as damage (Coufal et al., 2011). Aicardi‐Goutiéres syndrome is characterized by mutations in genes that inhibit reverse transcription (Zhao et al., 2013; Upton et al., 2015). It is currently unclear how LINE1 contributes mechanistically to these diseases, but it may involve disruption of normal gene transcription.

Rare CNVs associated with disease are supported by somatic repeat expansions that have been reported for multiple pathological states, as seen in tissue‐specific CAG repeat expansion profiles in Huntington's Disease (Telenius et al., 1994; La Spada, 1997; Shelbourne et al., 2007; Gonitel et al., 2008; Kraus‐Perrotta and Lagalwar, 2016). The highest levels of repeat length instability are observed in the brain, predominantly in neurons of brain regions most severely affected by the disease (Telenius et al., 1994; Shelbourne et al., 2007; Gonitel et al., 2008). Repeat expansion GM has been additionally implicated in spinocerebellar ataxia (La Spada, 1997; Kraus‐Perrotta and Lagalwar, 2016), frontotemporal dementia, amyotrophic lateral sclerosis (Almeida et al., 2013), and dentatorubral‐pallidoluysian atrophy (Ueno et al., 1995). Interestingly, somatic repeat expansion variation may occur in postmitotic neurons, supporting neural GM disease mechanisms in both mitotic and postmitotic periods (Gonitel et al., 2008; Kraus‐Perrotta and Lagalwar, 2016), and consistent with DCV changes that are most prominent during adult life (Westra et al., 2010; Bushman et al., 2015). GM produced by SNVs has been linked to rare brain diseases like hemimegalencephaly and focal cortical dysplasia involving point mutations in MTOR pathway genes (Evrony et al., 2012; D'Gama et al., 2017).

Beyond rare familial brain disorders, neural GM has been linked to sporadic Alzheimer's disease (AD) through both increased DCV and specific CNV amplification of the pathogenic gene, Amyloid Precursor Protein (*APP*). DCV increases of ∼200 Mb over the normal 250 Mb within prefrontal cortical neurons indicate significant, subgenomic increases in DNA content that are not explained by cell cycle reentry (Yang et al., 2001; Westra et al., 2009) nor trisomy 21 (Heston and Mastri, 1977; Potter, 1991) in view of more recent reports (Westra et al., 2009; Bushman et al., 2015). Most notably, single neuron qPCR for *APP* combined with PNA‐FISH for proximal and distal *APP* exons identified increased *APP* copy numbers of up to 12 copies, arising somatically and mosaically in sporadic AD neurons (Bushman et al., 2015): CNV increases of just 3 *APP* copies is pathogenic for AD in Down syndrome (via trisomy 21 on which *APP* resides) (Wiseman et al., 2015) and rare familial cases of *APP* locus duplication (Hooli et al., 2012). These results suggest a more general paradigm for neurological and neuropsychiatric sporadic brain disease, whereby known genes from rare, familial cases—such as *APP* for Down syndrome or familial AD—are somatically and mosaically altered by GM to produce common forms of disease. This same model may play out in other genomic regions whose germline alterations are not compatible with life—and therefore have not been identified in familial disease—but may be altered mosaically to produce sporadic forms of a disease.

## CONCLUDING COMMENTS

**Figure 7:**
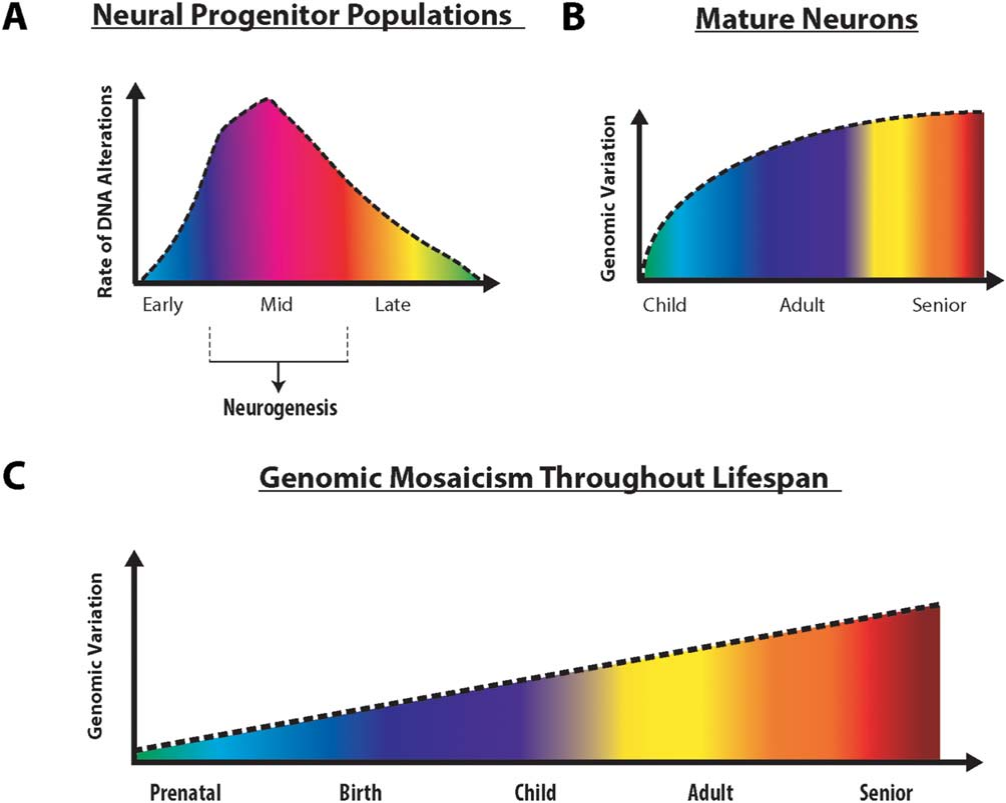
Rate of DNA alteration in developing brain and distribution of genomic mosaicism in mature neurons. **(A)** In embryonic brain development, DNA alteration rates peak during periods corresponding with high levels of neurogenesis, before leveling off. **(B)** After prenatal development, genomic alterations continue to accumulate during neonatal and childhood neurodevelopmental periods, with smaller alterations accruing thereafter. **(C)** Taken together, genomic mosaicism accumulates over a lifetime, starting during embryonic brain development, and continuing throughout life and into adulthood and old age, potentially contributing to age related neurological disorders. Importantly, the vast majority of these somatic changes can only be detected when the mosaic nature of individual neurons is considered in experimental design. [Color figure can be viewed at http://wileyonlinelibrary.com]

Over the last 20 years, neural GM has advanced from a theoretical concept to a definitive experimental fact and now represents a vibrant field of active research. The proven forms of GM within single cells of the brain—aneuploidies and aneusomies, other CNVs, and SNVs—are no doubt the “tip of the iceberg” in considering the pervasive presence of DCV throughout the brain that captures virtually all forms of DNA sequence alterations, affecting both mitotic and postmitotic populations. The combination of these alterations contribute to increased GM over time (Fig. 7a–c). As perhaps the most stable and long lasting biological substrate within the brain, DNA changes produced by GM may underlie fundamental brain activities including complex behaviors and long term memory. The presence of DNA fragmentation and double strand breaks amongst developing brain cell populations associated with cell death and differentiation likely involves recurrent breaks in specific genes, as reported from studies of neural progenitor cell populations (Wei et al., 2016), which are again reminiscent of processes in the adaptive immune system (Chun, 2001; Kingsbury et al., 2006; Westra et al., 2010; Bushman and Chun, 2013). It would thus not be surprising to find novel forms of DNA rearrangement within cells of the brain, given the postmitotic state of neurons (distinct from clonally expanded alterations of the immune system) and expression of different genes (e.g., *RAG1* but not *RAG2* within the brain vs. both in the immune system). All combined, these diverse, nonmutually exclusive and pervasive forms of neural GM could “barcode” each brain cell by creating a unique genome, thus representing a small universe of genome diversity residing within a single brain. Moreover, further evidence that this universe changes over time adds another dimension of complexity, representing a relatively unassessed variable contributing to neural diversity at all levels of brain development and function. This same genomically diverse universe is currently unrecognized by virtually all genetic models of brain disease, particularly those relying on statistical relationships of genes identified from cells outside of the brain, as is common for genome‐wide association studies (GWAS). The overwhelming prevalence of sporadic brain disease unaccounted for by defined familial genes—as observed in Alzheimer's disease—may be more fully explained by mosaic genomic changes that affect both the genes identified in rare familial cases, as well as new genes and nongenic loci (including mutations that may not be compatible with life if present constitutively), particularly within postmitotic neurons. Individual cells altered in sporadic disease could offer a rich, new source for discovery of meaningful disease targets. GM within the brain therefore represents a vast frontier awaiting further exploration and discovery, toward more fully understanding the developing and functioning brain and its diseases.
